# Do Targeted Bans of Insecticides to Prevent Deaths from Self-Poisoning Result in Reduced Agricultural Output?

**DOI:** 10.1289/ehp.11029

**Published:** 2008-01-22

**Authors:** Gamini Manuweera, Michael Eddleston, Samitha Egodage, Nick A. Buckley

**Affiliations:** 1 Office of the Pesticide Registrar, Government Department of Agriculture, Peradeniya, Sri Lanka; 2 South Asian Clinical Toxicology Research Collaboration, Department of Clinical Medicine, University of Colombo, Colombo, Sri Lanka; 3 Centre for Tropical Medicine, Nuffield Department of Clinical Medicine, University of Oxford, Oxford, United Kingdom; 4 Clinical Pharmacology and Toxicology, Australian National University Medical School, Canberra, Australian Capital Territory, Australia

**Keywords:** food production, pesticide poisoning, pesticide regulation, public health policy, suicide prevention

## Abstract

**Background:**

The pesticides monocrotophos, methamidophos, and endosulfan were a very common cause of severe poisoning in Sri Lanka during the 1980s and early 1990s, before they were banned in 1995 and 1998. Now, the most commonly used insecticides are the less toxic World Health Organization Class II organophosphorus pesticides and carbamates. These bans were followed by a large reduction in both fatal poisonings and suicide in Sri Lanka.

**Objective:**

We aimed to see if these bans adversely affected agricultural production or costs.

**Methods:**

We used data from the World Resources Institute to compare the yields of the main crop groups in Sri Lanka with those from surrounding South Asian countries for 1980–2005. We also examined data from the Sri Lankan Department of Census and Statistics to examine the yields of 13 specific vegetable crops and rice for 1990–2003, along with the costs of rice production.

**Results:**

We found no drop in productivity in the years after the main bans were instituted (1995, 1998). We observed substantial annual fluctuation in estimated yields in all data sources, but these did not coincide with the bans and were no larger than the fluctuations in other countries. Also, there was no sudden change in costs of rice production coinciding with bans.

**Conclusions:**

Countries aiming to apply restrictions to reduce deaths from pesticide poisoning should evaluate agricultural needs and develop a plan that encourages substitution of less toxic pesticides. If farmers have an affordable alternative for pest control for each crop, there is no obvious adverse effect on agricultural output.

Pesticide self-poisoning is a major problem in rural areas of the Asian Pacific developing world (Eddleston and Phillips 2004; Konradsen et al. 2005). Widespread agricultural use of pesticides and home storage make them easily available for acts of self-harm in many rural households. Some clinicians have called for bans of particular pesticides that cause major local problems (Daisley and Hutchinson 1998; Siwach and Gupta 1995). Others within both the clinical and agricultural communities have called for the removal of all highly toxic pesticides on public health grounds [Eddleston et al. 2002; Food and Agriculture Organization of the United Nations (FAO) Committee on Agriculture 2007; Konradsen et al. 2003]. However, some agronomists and the pesticide industry have warned that such bans may adversely affect agricultural output or prices in the affected regions (Cooper and Dobson 2007; Oerke and Dehne 2004; Oerke et al. 1994). Thus far, however, there have been no studies to support or refute these assertions.

The problem of pesticide self-poisoning in the context of high suicide rates has been recognized at the highest levels in Sri Lanka. The World Health Organization (WHO) Class I toxicity organophosphorus pesticides (OP) were a very common means of suicide in the 1980s and early 1990s (Roberts et al. 2003; Van der Hoek and Konradsen 2006). Parathion and methylparathion were banned in the mid-1980s, and the last of the Class I OPs, monocrotophos and methamidophos, were banned in 1995 (Roberts et al. 2003; Van der Hoek and Konradsen 2006). Unfortunately, their place in the market was taken by endosulfan, resulting in an epidemic of self-poisoned patients with status epilepticus and many deaths (Roberts et al. 2003). This insecticide in turn was banned in 1998, and the current most commonly used insecticides are the WHO Class II OPs and carbamates (Roberts et al. 2003; Van der Hoek and Konradsen 2006).

In a previous study (Roberts et al. 2003) carried out in the hospitals of the North Central Province (NCP) of Sri Lanka, we showed that these bans resulted in these three pesticides being no longer the cause of any poisonings within a few years. Although the number of pesticide poisonings presenting to the hospital did not decrease, there was a significant reduction in the total number of poisoning deaths. We have also shown there has been a 40–50% progressive reduction in suicide by self-poisoning with pesticides and in the overall suicide rate over 1995–2002; these two bans are the most plausible explanation (Figure 1) (Gunnell et al. 2007).

These national public health pesticide bans offer the opportunity to observe the effects of such bans on agricultural output. We therefore investigated whether changes in production and costs for major crops have occurred during the period of these bans; other countries in South Asia have similar problems with very high rates of suicide due to insecticides, but they have not instituted bans. We also wished to compare longitudinal trends in Sri Lankan agricultural production with that in the neighboring countries.

## Methods

### Sources of data

We obtained longitudinal whole-country data on agricultural production and yields for the South Asian countries of Sri Lanka, India, Pakistan, and Bangladesh from the World Resources Institute (2007) website. These data are collated from data supplied by these countries to the FAO.

Longitudinal data on a range of specific crops for the whole of Sri Lanka was obtained from Sri Lanka’s Department of Census and Statistics (2007a). These data are based on regular surveys conducted by the Ministry of Agriculture.

Paddy rice is the most important agricultural product in Sri Lanka (International Rice Research Institute 2007). To further explore the range of possible factors involved in altering production and costs, we obtained longitudinal data for the NCP on paddy productivity, costs, and consumer price index movements. These came from the Department of Census and Statistics (2007) and the Ministry of Agriculture database through the Hector Kobbekaduwa Agrarian Research and Training Institute (Colombo, Sri Lanka). The Ministry of Agriculture estimates are based on annual field surveys.

## Results

**Figure 2:**
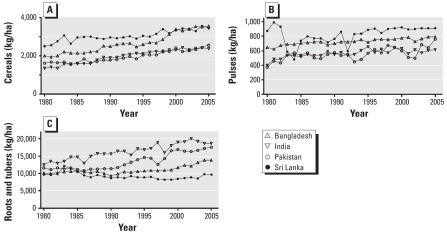
Yields of cereals (*A*), pulses (*B*), and roots and tubers (*C*) in south Asian countries during 1980–2005.

The yields of the main crop groups in Sri Lanka showed no obvious drop in productivity in the years after the main bans were instituted (1995 and 1998). There was substantial annual fluctuation in estimated yields, but these did not coincide with the bans and were no larger than the fluctuations in other countries. On average, the Sri Lankan yields for cereals and pulses are higher and those for roots and tubers are lower than those of the neighboring countries (Figure 2).

**Figure 3:**
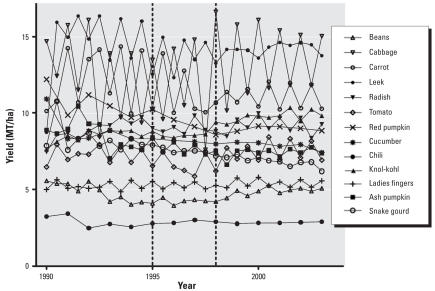
Longitudinal changes in Sri Lankan yields of 13 vegetable crops during 1990–2004. MT, metric tons. Vertical lines indicate the years when all Class I insecticides (1995) and endosulfan (1998) were banned.

The data on the Sri Lankan yields of 13 vegetable crops during 1990–2003 also show no obvious drops in productivity at the time of the bans (Figure 3). We did observe some downward trends in cucurbit (pumpkins, cucumber, and gourds) vegetable production that predate these bans, but otherwise, production yields have been remarkably constant, with the obvious seasonal variation due to the different monsoonal rainfall with the Maha and Yala seasons. We examined the production of tea, rubber, and coconut, and these also did not change during this time (data not shown).

**Figure 4:**
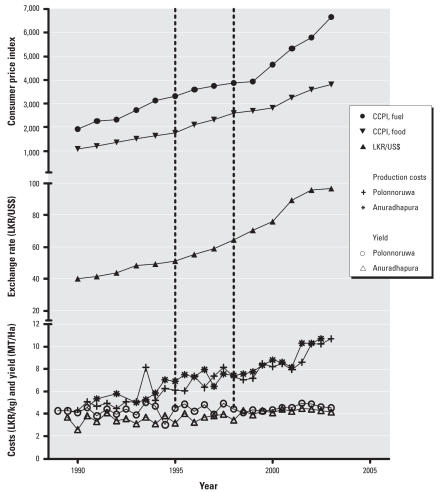
Longitudinal changes in paddy production and costs of production in two districts (Anuradhapura and Polonnaruwa) of the NCP and correlation with external major cost items. Abbreviations: CCPI, Colombo Consumer Price Index; LKR, Lanka rupees; MT, metric tons. Vertical lines indicate the years when all Class I insecticides (1995) and endosulfan (1998) were banned.

Rice paddy production varied with the Maha and Yala seasons, but we saw no other apparent change at the time of the bans (Figure 4). Production costs for paddies within the NCP have increased steadily over time. However, there was no change in this rate that coincided with the bans; in contrast, a significant increase in production costs occurred because of rising fuel prices and deterioration of the exchange rate around 2002–2003. We examined the production costs of a number of other crops (data not shown), none of which showed a change in the trend at this time.

## Discussion

In the present study, we found no good evidence that a pesticide ban necessarily results in reduced output or increased costs to the farmer. Overall, we found no significant change in food production during the 1990s, and no change in the rate of increase in production costs or yield that could be attributed to the pesticide restrictions.

The pesticides were targeted on the basis of Ministry of Health data indicating that specific insecticides were of concern because of large numbers of poisoning deaths. However, the bans of specific insecticides were coordinated by the Ministry of Agriculture. Therefore, the needs of farmers to have an affordable insecticide for pest control for each crop were taken into account. Before the regulations, in 1988–1990, monocrotophos and methamidaphos were widely used. They accounted for 60–75% of the total volume of OPs imported each year (Ministry of Agriculture, unpublished data). These two OPs were also approved for use on a wide variety of crops, and yet their bans led to no obvious adverse effect on agricultural output of any single crop. For each crop and pest, a number of other affordable pesticides with equivalent activity were approved and available for use.

Rice is the most important crop in Sri Lanka (International Rice Research Institute 2007). Our results are consistent with findings of integrated pest management (IPM) programs, indicating that the need for insecticides in rice is often overestimated. These programs in Sri Lanka have lead to large reductions in total pesticide usage and increased yields (Van den Berg et al. 2002), and similarly favorable impacts of IPM have been found for many other crops in other countries (Van den Berg 2004). Studies of rice yield in Sri Lanka have identified that production is largely determined by water supply, nutrient content of the soil, and cultivar. Losses due to pests are not regarded as an important determinant of yield in recent decades after the green revolution because of improved cultivars and the use of pesticides (Dhanapala 2007). However, to investigate minor changes in production or costs related to pesticide regulation would require prospective agricultural studies that carefully control for these other factors that together largely determine rice yields (e.g., a study performed by a national rice research center).

The problem of pesticide poisoning is widespread within the region. Other countries aiming to apply pesticide restrictions to reduce poisoning incidents and deaths should bear in mind the needs of agriculture in order to be accepted and receive cooperation from the local communities. An increase in production costs might have occurred if there had been enforced use of more expensive or less-efficient pesticides. However, in the present study, the only correlation was with worsening foreign exchange rates that made imports more expensive, particularly fuel prices. The 2.5-fold increase in production costs for paddies is in line with the 2.5-fold deterioration in the exchange rate and the 3.5-fold increase in the Sri Lankan cost of living index during 1990–2003. Again, this is not surprising because the price of the banned pesticides was not less than that of comparable OPs in this case. Moreover, agrochemicals such as fertilizer and pesticides are currently a relatively small component of the total costs of rice production compared with labor and fuel, which together account for > 75% of total production costs (International Rice Research Institute 2007). However, regulatory restrictions to shift agricultural use to newer, less toxic non-anticholinesterase insecticides might lead to significant price increases. Studies are now being undertaken to examine the economics and impact of this strategy. There may be additional health benefits from reduced acute and chronic occupational poisoning and therefore other economic benefits because of greater productivity; however, these benefits are far more difficult to quantify.

The initial pesticide bans were largely based on a simple strategy of phased removal of all Class I (extremely hazardous) pesticides in Sri Lanka. This is similar to the global strategy now proposed by the FAO Committee on Agriculture (2007). However, the use of public health data was critical in identifying the specific Class II (moderately hazardous) pesticide that was of more concern (endosulfan). We have recently identified two other Class II OPs with relative greater human toxicity (Eddleston et al. 2005). It will be important to evaluate agricultural needs and a strategy for substitution in developing a regulatory strategy to further reduce deaths.

## Figures and Tables

**Figure 1:**
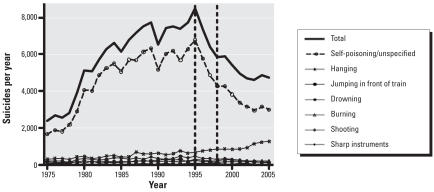
Change in suicide rate and deaths from poisoning between 1975 and 2005. Vertical lines indicate the years when all Class I insecticides (1995) and endosulfan (1998) were banned. Adapted from Gunnell et al. (2007).
